# Immune interaction between *Aspergillus fumigatus* and non-tuberculous mycobacteria

**DOI:** 10.3389/fcimb.2026.1810773

**Published:** 2026-05-04

**Authors:** Hotaka Namie, Takahiro Takazono, Satoshi Irifune, Yuya Ito, Nana Nakada, Tatsuro Hirayama, Masataka Yoshida, Kazuaki Takeda, Shotaro Ide, Naoki Iwanaga, Masato Tashiro, Naoki Hosogaya, Noriho Sakamoto, Akira Watanabe, Yoshimasa Tanaka, Katsunori Yanagihara, Tomoya Nishino, Hiroshi Mukae, Koichi Izumikawa

**Affiliations:** 1 Department of Infectious Diseases, Nagasaki University Graduate School of Biomedical Sciences, Nagasaki, Japan; 2 Department of Respiratory Medicine, Nagasaki University Hospital, Nagasaki, Japan; 3 Health Center, Nagasaki University, Nagasaki, Japan; 4 Department of Pharmacotherapeutics, Nagasaki University Graduate School of Biomedical Sciences, Nagasaki, Japan; 5 Infectious Diseases Experts Training Center, Nagasaki University Hospital, Nagasaki, Japan; 6 Department of Infectious Disease Medicine, Yokohama City University Graduate School of Medicine, Yokohama, Japan; 7 Clinical Research Center, Nagasaki University Hospital, Nagasaki, Japan; 8 Division of Clinical Research, Medical Mycology Research Center, Chiba University, Chiba, Japan; 9 Center for Medical Innovation, Nagasaki University, Nagasaki, Japan; 10 Department of Laboratory Medicine, Nagasaki University Hospital, Nagasaki, Japan; 11 Department of Nephrology, Nagasaki University Hospital, Nagasaki, Japan; 12 Infection Control and Education Center, Nagasaki University Hospital, Nagasaki, Japan

**Keywords:** disease progression, lung diseases, macrophages, *Mycobacterium avium*, non-tuberculous mycobacteria, phagocytosis, pulmonary aspergillosis

## Abstract

**Introduction:**

The global prevalence of non-tuberculous mycobacterial pulmonary disease (NTM-PD) is increasing. Individuals with NTM-PD frequently develop chronic pulmonary aspergillosis, which is associated with poor clinical outcomes. However, the biological mechanisms underlying the interaction between *Aspergillus* species and non-tuberculous mycobacteria (NTM) remain poorly understood. This study aimed to investigate the interaction between *Aspergillus fumigatus* and NTM and to identify mechanisms that may inform novel therapeutic strategies.

**Methods:**

*A. fumigatus* was incubated with NTM culture supernatants, and biofilm formation was quantified using the crystal violet assay. Phagocytic activity against *A. fumigatus* conidia were assessed in THP-1-derived macrophages infected with *Mycobacterium avium*. Conversely, phagocytosis of *M. avium* was evaluated in macrophages exposed to *A. fumigatus* culture supernatants. Finally, fungal clearance *in vivo* was assessed in mice pre-infected with *M. avium*.

**Results:**

NTM supernatants significantly enhanced *A. fumigatus* growth. *M. avium* infection decreased the macrophages-phagocytosis rate of *A. fumigatus* by approximately 40% compared to uninfected control. Additionally, *M. avium* infection reduced Dectin-1 gene expression in macrophages by half. Secondary metabolites produced by *A. fumigatus* impaired macrophage phagocytosis of *M. avium*. *In vivo*, prior *M. avium* infection delayed fungal clearance from the lungs.

**Discussion:**

NTM promote not only *A. fumigatus* growth but also its colonization by impairing macrophage immune function. Conversely, *A. fumigatus* suppresses host defense against NTM via secondary metabolites. These findings suggest that microbial cross-modulation creates a permissive niche that facilitates co-colonization and may contribute to disease progression.

## 1 Introduction

*Aspergillus* species are ubiquitous environmental fungi that cause respiratory disease following the inhalation of airborne conidia. *Aspergillus fumigatus* is the predominant pathogen responsible for pulmonary aspergillosis (Pagano et al., 2010; Tashiro et al., 2011), and the clinical manifestations range from colonization to invasive disease depending on the host immune status. Individuals with hematological malignancies or those receiving intensive chemotherapy are at particularly high risk of invasive pulmonary aspergillosis, which carries a reported mortality of 32–90% (Lin et al., 2001; Henao-Martínez et al., 2023; Feng et al., 2025). Current clinical guidelines recommend long-term antifungal therapy for at least 6 months for the management of chronic pulmonary aspergillosis (CPA) (Denning et al., 2016). However, treatment is frequently limited by adverse effects associated with prolonged antifungal exposure (Benitez and Carver, 2019), underscoring the need for alternative therapeutic strategies.

Chronic obstructive pulmonary disease (COPD), previous pulmonary tuberculosis, and non-tuberculous mycobacterial pulmonary disease (NTM-PD) are common comorbidities in patients with CPA. Even among individuals with advanced COPD, the prevalence of CPA is estimated at 0.3–9.8% (Gu et al., 2021; Palanivel et al., 2025); in contrast, a higher prevalence of 3.5–16.7% has been reported in patients with NTM-PD (Zoumot et al., 2014; Tanaka et al., 2025). Retrospective studies regarding co-infections of non-tuberculous mycobacteria (NTM) and *Aspergillus* have indicated that the NTM infection often precedes the onset of CPA or diagnosed at the same time (Takeda et al., 2016; Takazono et al., 2025). Notably, patients with NTM-PD complicated by CPA experience significantly worse outcomes compared with those without CPA (Takeda et al., 2016; Takazono et al., 2025), and NTM-PD itself has been identified as an independent prognostic factor for poor survival in CPA (Lowes et al., 2017). Given the increasing global incidence and mortality associated with NTM-PD, cases of dual infection with CPA are expected to rise.

Previous studies have explored microbial interactions between *Aspergillus* spp. and several bacterial pathogens, including *Klebsiella pneumoniae*, *Streptococcus pneumoniae*, and *Stenotrophomonas maltophilia* (Melloul et al., 2016; Iwahashi et al., 2020; Curtis et al., 2023). Clinically, co-infection with *A. fumigatus* and *Pseudomonas aeruginosa* is well recognized in individuals with cystic fibrosis, and mechanisms of microbial competition and cooperation have been proposed (Keown et al., 2020). In contrast, data on interactions between *A. fumigatus* and NTM are scarce, and the immunological mechanisms remain undefined. In this study, we aimed to investigate the bidirectional interaction between *A. fumigatus* and NTM to clarify why CPA frequently complicates NTM-PD and to generate mechanistic insights that may support the development of novel therapeutic approaches. We hypothesized that NTM promotes *Aspergillus* colonization, while conversely, *Aspergillus* suppresses the host immune response against NTM.

## 2 Material and methods

### 2.1 Strains and cell preparation

*A. fumigatus* strain Af293 was used as the reference strain. A Δ*laeA* mutant derived from Af293, lacking the *laeA* gene and therefore deficient in secondary metabolite production (Bok and Keller, 2004), was obtained from the Department of Medical Microbiology and Immunology, University of Wisconsin–Madison. *A. flavus* (NBRC6343), *A. niger* (NBRC10564), and *A. terreus* (NBRC6346) were used as *Aspergillus* spp. other than *A. fumigatus*. *Mycobacterium avium* (ATCC 700737) and *M. abscessus* (ATCC 19977) were used as representative NTM, and *P. aeruginosa* strain PAO1 was included for comparison. All fungal conidia and bacterial isolates were stored at −80 °C. Fungal conidia were cultured on potato dextrose agar (PDA; Becton Dickinson, Franklin Lakes, NJ, USA) at 37 °C for 4–7 days. Hyphal fragments were removed using a 40 μm cell strainer, after which conidia were pelleted (3,000 rpm, 5 min), washed in phosphate-buffered saline (PBS), and resuspended in Roswell Park Memorial Institute (RPMI) 1640 medium (FUJIFILM Wako, Osaka, Japan). Swollen conidia were generated by shaking the cultures in RPMI medium supplemented with 10% fetal bovine serum (FBS) at 37 °C and 250 rpm for 7 h, followed by heat inactivation at 95 °C for 20 min.

THP-1 monocyte cells (ATCC, Manassas, VA, USA) were maintained in RPMI-1640 medium containing 10% FBS at 37 °C with 5% CO_2_. Cells were seeded at 2.0 × 10^6^ cells/mL (0.5 mL/well in 24-well plates or 2.5 mL/well in 6-well plates) and differentiated into macrophages using 100 nM phorbol 12-myristate 13-acetate (Sigma-Aldrich, St. Louis, MO, USA) for 24 h. Differentiated THP-1 macrophages were infected with *M. avium* at a multiplicity of infection (MOI) of 10 for 3 h. After infection, extracellular bacteria were removed by washing with PBS, and cells were cultured for additional 24 h in RPMI-1640 medium. Intracellular antibacterial treatment, when required, was performed for 24 h using clarithromycin-supplemented RPMI medium (Tokyo Chemical Industry, Tokyo, Japan).

### 2.2 Supernatant preparation

NTM were sub-cultured twice on Middlebrook 7H10 agar (Becton Dickinson) and colonies were suspended in RPMI-1640 medium. This was then statically incubated for 1 week. Following centrifugation, the bacterial suspension was resuspended in fresh RPMI-1640 medium and stored at −80 °C. To prepare NTM supernatants, NTM suspension was adjusted to a concentration of 1.3 × 10^8^–4.0 × 10^8^ CFU/mL in RPMI medium. The suspension was then shaken at 37 °C and 250 rpm for 1 week. *Pseudomonas aeruginosa* (PAO1) was incubated overnight in Luria–Bertani medium (Becton Dickinson) at 37 °C and 250 rpm. PAO1 was then resuspended in RPMI medium and adjusted to an optical density (OD) of 0.1 at 600 nm. The bacterial suspension was cultured at 37 °C and 250 rpm for 24 h. *Aspergillus fumigatus* conidia were adjusted in RPMI medium at a final concentration of 1.0 × 10^7^ conidia/mL, applied at 500 μL per well in 24-well plates. The fungal suspension was then cultured at 37 °C with 5% CO_2_ for 48 h. All supernatants were centrifuged and filtered through 0.22 µm membrane filters to remove bacteria or hyphal fragments and then stored at −20 °C until use.

### 2.3 Crystal violet biofilm assay

*A. fumigatus* and other *Aspergillus* spp. conidia were suspended in RPMI-1640 medium and adjusted to 1.0 × 10^5^ conidia/mL. A 100 μL aliquot of the suspension was added to each well of a flat-bottomed 96-well plate, followed by 100 μL of NTM or *P. aeruginosa* culture supernatant. Plates were incubated at 37 °C with 5% CO_2_ for 48 h to allow biofilm formation. *A. terreus* was incubated for 120 h due to its slow growth speed. Following incubation, biofilms were stained with 0.1% crystal violet (Sigma-Aldrich) (100 μL/well, 10 min), washed twice with PBS, and destained using 99.5% ethanol (125 μL/well). A 75 µL aliquot of the resulting solution was transferred to a fresh plate, and optical density (OD) was measured at 595 nm using a 620 nm reference wavelength to quantify biofilm biomass.

### 2.4 Microscopy of biofilms

#### 2.4.1 Confocal laser scanning microscopy

Biofilms prepared using the same method described in the crystal violet assay were fixed with Mildform 10N (FUJIFILM Wako) and stained with calcofluor white (Sigma-Aldrich) at 25 °C for 10 min. After staining, samples were washed twice with PBS and visualized using a confocal laser scanning microscope (BZ-X700, Keyence, Osaka, Japan). To evaluate the effect of *M. avium* infection on the immune response of macrophages against *A. fumigatus*, *M. avium*-infected THP-1 cells were co-cultured with live *A. fumigatus* conidia at an MOI of 0.3 in RPMI medium without FBS. After 24 hours of co-culture, *A. fumigatus* hyphae were fixed overnight in Mildform 10N and stained with calcofluor white at 25 °C for 10 minutes. Four well samples were prepared for each condition, and four corners were captured per well at 40x magnification. The area of fluorescent hyphae was quantified using the hybrid cell count function in the associated image analysis software (BZ-X Analyzer, Keyence).

#### 2.4.2 Transmission electron microscopy

A conidial suspension of *A. fumigatus* (150 μL per well) was inoculated onto sterile cover glass placed in a 24-well plate. A further 150 μL of NTM supernatant was added, yielding a final concentration of 1.0 × 10^6^ conidia/mL. Fixation and imaging procedures were performed as described in the Supplementary Methods, and biofilm structure on the cover glass was evaluated by TEM. Five images were captured from each sample, and images showing hyphae of similar diameter were selected to measure the length of the extracellular matrix.

### 2.5 Quantitative polymerase chain reaction

Swollen *A. fumigatus* conidia were added to THP-1 macrophages previously infected with *M. avium* and incubated for 6 h at an MOI of 2. Total RNA was extracted using the RNeasy Plus Kit (QIAGEN, Hilden, Germany), and cDNA was synthesized using the QuantiTect Reverse Transcription Kit (QIAGEN). qPCR reaction was performed using Applied Biosystems QuantStudio 12K Flex platform under the following cycling conditions: initial denaturation at 95 °C for 15 min, followed by 40 cycles of 94 °C for 30 s, 42 °C for 30 s, and 72 °C for 35 s. Gene targets included *Dectin-1*, Toll-like receptor 2 (*TLR-2*), and NADPH oxidase 2 (*NOX2*), which are key components of host antifungal immunity (Gantner et al., 2003) (Philippe et al., 2003). Primer sequences are listed in **Supplementary Table 1**. Relative gene expression was normalized to glyceraldehyde-3-phosphate dehydrogenase (*GAPDH*). Fold changes in mRNA expression were calculated using the 2^ΔCT^ method, where ΔCt = (Ct_gene – Ct_*GAPDH*).

### 2.6 Flow cytometer analysis

THP-1 macrophages previously infected with *M. avium* were co-cultured with calcofluor-white- labeled *A. fumigatus* conidia for 2 h at an MOI of 2. This experiment was performed to assess whether NTM infection alters the phagocytic activity of THP-1 cells toward *A. fumigatus*. Surface expression of the pattern-recognition receptor Dectin-1 on THP-1 cells following NTM exposure was also evaluated by flow cytometry. To investigate the effect of fungal secondary metabolites, THP-1 cells exposed to *A. fumigatus* culture supernatant or gliotoxin (Sigma-Aldrich) were co-cultured with Alexa Fluor 647-labeled *M. avium* for 3 h at an MOI of 10. This assay was designed to determine whether *A. fumigatus*-derived metabolites influence macrophage phagocytosis of NTM. Detailed staining conditions and gating strategies are provided in the Supplementary Methods. All flow cytometer analyses were performed using the FACS Lyric flow cytometer (Becton Dickinson) and FlowJo ver. 10 (FlowJo LLC, Ashland, OR). The antibodies used were as follows: Fluorescein isothiocyanate (FITC)-conjugated anti-CD11c monoclonal antibody(mAb) (Clone: Bu15, Biolegend, San Diego, CA, USA), Allophycocyanin (APC)-conjugated anti-Dectin-1/CLEC7A mAb (Clone; 15E2, Biolegend).

### 2.7 *In vivo* infection model

Female BALB/cCrSlc mice aged 7 weeks (Japan SLC, Shizuoka, Japan) were intratracheally inoculated with *M. avium* at a dose of 2.5 × 10^7^ colony-forming units (CFU) per mouse on Days 0 and 14 (Co-infection group). *A. fumigatus* infectionmice received an equivalent volume of PBS. To ensure prior infection, *M. avium* was administered twice. The mice were housed for approximately two months, after which lung tissue destruction progressed due to *M. avium* infection. On Day 77, both groups were intratracheally inoculated with *A. fumigatus* conidia (5 × 10^6^ conidia per mouse). Lung fungal burden was assessed 1 day, 1 week, and 2 weeks after *A. fumigatus* challenge. Harvested lungs were homogenized, serially diluted in PBS; 50 μL of the diluted solution was then plated onto PDA medium, and CFUs were quantified 24 hours later. All animal experimental procedures were approved by the Committee for Animal Experimentation and Use at Nagasaki University (approval no. 2109091745-138).

### 2.8 Statistical analyses

Statistical analyses were performed using GraphPad Prism (version 9.3.1). Group differences were evaluated using unpaired Student’s *t*-tests, one-way analysis of variance (ANOVA) with Tukey’s *post-hoc* test, or two-way ANOVA with Sidak’s *post-hoc* test. A *P*-value of < 0.05 was considered statistically significant. Data under the same conditions were treated as technical replicates *in vitro* experiments. To assess reproducibility, *in vitro* experiments were independently repeated at least three times, whereas *in vivo* experiments were repeated twice.

## 3 Results

### 3.1 NTM supernatants promote *A. fumigatus* biofilm formation

**Figure 1:**
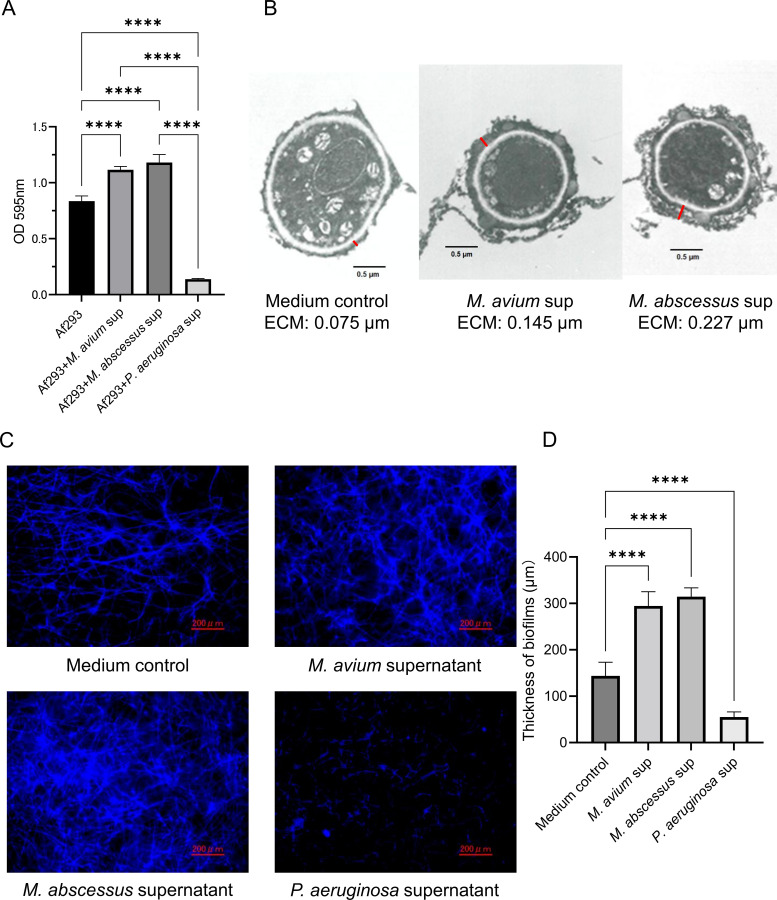
Effect of non-tuberculous mycobacteria (NTM) supernatants on *Aspergillus fumigatus* biofilm formation. **(A)** Quantification of *A. fumigatus* biofilm biomass using the crystal violet assay after exposure to supernatants from *Mycobacterium avium*, *M. abscessus* or *Pseudomonas aeruginosa*. Supernatants from *M. avium* and *M. abscessus* increased biofilm formation, whereas *P. aeruginosa* supernatant reduced biomass (****P < 0.0001). Bar graph shows the mean and standard deviation of technical replicates (*n* = 5). Data represent at least three independent experiments. **(B)** Transmission electron microscopy images of *A. fumigatus* biofilms showing extracellular matrix (ECM) distribution. ECM thickness was measured in regions with uniform matrix appearance (red measurement lines). Scale bar = 0.5 µm. **(C)** Confocal laser scanning microscopy (CLSM) images of calcofluor white-stained *A. fumigatus* biofilms. Scale bar = 200 µm. **(D)** Quantification of biofilm thickness Based on Z-stack measurements from the well surface to the uppermost biofilm layer (****P < 0.0001). Bar graph shows the mean and standard deviation of technical replicates (*n* = 6). Data represent at least three independent experiments.

Exposure to culture supernatants from *M. avium* and *M. abscessus* significantly increased *A. fumigatus* biofilm biomass compared with medium control (**Figure 1A**). TEM revealed greater extracellular matrix (ECM) deposition surrounding the fungal hyphae in the presence of NTM supernatants (**Figure 1B**). CLSM further confirmed enhanced biofilm architecture and significantly increased biofilm thickness following exposure to NTM supernatants (**Figures 1C, D**). A similar biofilm-enhancing effect was observed in other *Aspergillus* spp. including *A. flavus*, *A. niger*, and *A. terreus* (**Supplementary Figure 1A**). The biofilm-promoting activity of NTM supernatants was retained following heat treatment and ultrafiltration, indicating the presence of a heat-stable, low molecular weight bioactive component (**Supplementary Figures 1B, C**). In addition, *A. fumigatus* biofilms formed in the presence of NTM supernatants displayed reduced susceptibility to voriconazole when compared with untreated controls (**Supplementary Figure 2**).

### 3.2 Supernatant from *A. fumigatus* does not alter NTM growth

NTM growth was evaluated by measuring the OD after treatment with *A. fumigatus* culture supernatant. No significant change in the growth of either *M. avium* or *M. abscessus* was observed following exposure to *A. fumigatus* supernatant (**Supplementary Figures 3A, B**).

### 3.3 *M. avium* infection impairs phagocytosis of *A. fumigatus* conidia by THP-1 macrophages

**Figure 2:**
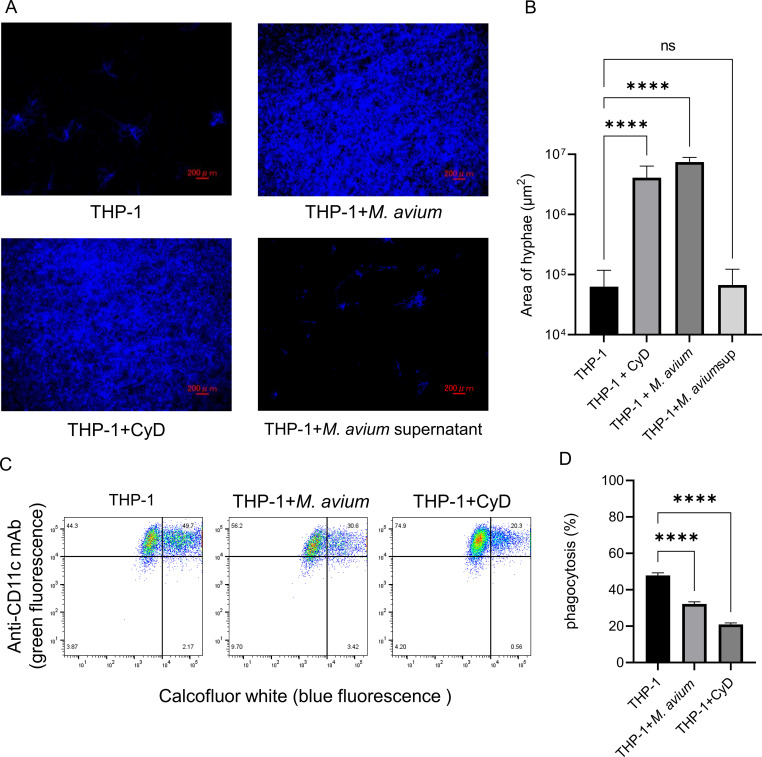
Effect of *Mycobacterium avium* infection on the antifungal response of THP-1 macrophages against *Aspergillus fumigatus*. **(A)** Confocal laser scanning microscopy (CLSM) images of calcofluor white-stained *A. fumigatus* hyphae following 24-h co-culture with THP-1 macrophages. THP-1 cells were pre-infected with *M. avium* at a multiplicity of infection (MOI) of 10 before exposure to resting *A. fumigatus* conidia (MOI 0.3). Cytochalasin D (CyD) was used as a positive control for inhibition of phagocytosis. Scale bar = 200 µm. **(B)** Quantification of hyphal area based on fluorescence imaging using hybrid cell count analysis (****P < 0.0001). Bar graph shows the mean and standard deviation of technical replicates (*n* = 16). Data represent at least three independent experiments. **(C)** Flow cytometry plots showing phagocytosis assay of calcofluor white-labeled *A. fumigatus* conidia by THP-1 macrophages. The upper-right quadrant represents the double-positive events corresponding to THP-1 cells that internalized conidia. **(D)** Quantification of phagocytosis rate. Infection with *M. avium* significantly reduced the proportion of THP-1 macrophages phagocytosing *A. fumigatus* conidia (****P < 0.0001). Bar graph shows the mean and standard deviation of technical replicates (*n* = 4). Data represent at least three independent experiments.

Representative fluorescent microscopy images show *A. fumigatus* hyphae following 24-h co-culture with THP-1 macrophages under each condition (**Figure 2A**). Quantification of hyphal area demonstrates a significant increase when THP-1 cells were pre-infected with *M. avium*, compared with uninfected controls (**Figure 2B**). In contrast, exposure to *M. avium* supernatant alone did not enhance hyphal expansion relative to control conditions. Flow cytometry analysis demonstrated a marked reduction in the proportion of THP-1 cells that internalized *A. fumigatus* conidia following *M. avium* infection (**Figures 2C, D**). Together, these findings indicate that *M. avium* infection suppresses macrophage-mediated phagocytosis of *A. fumigatus*, allowing *A. fumigatus* to escape from immune responses of THP-1 cells and extend its hyphae. Whereas soluble factors in *M. avium* supernatant alone did not affect the antifungal activity of THP-1 cells against *A. fumigatus*.

### 3.4 Antimycobacterial treatment restores phagocytic control of *A. fumigatus* in *M. avium*-infected THP-1 cells

**Figure 3:**
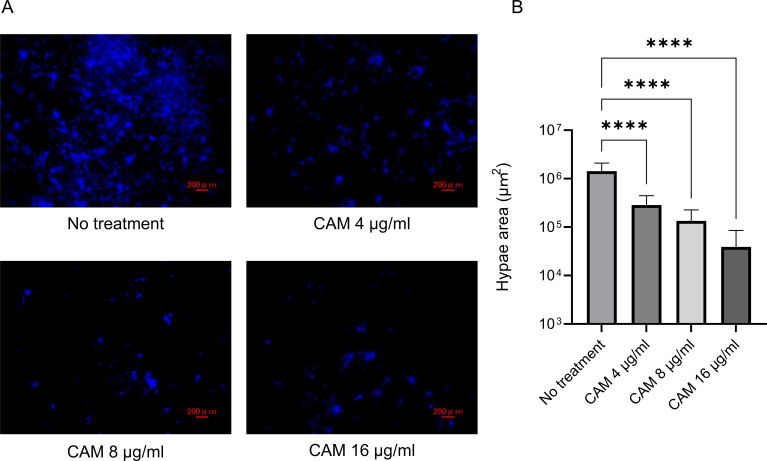
Effect of clarithromycin (CAM) treatment on antifungal activity of THP-1 macrophages infected with *Mycobacterium avium*. **(A)** Confocal laser scanning microscopy images of calcofluor white-stained *Aspergillus fumigatus* hyphae following co-culture with THP-1 macrophages treated with CAM. THP-1 cells first infected with *M. avium* and then exposed to 4–16 μg/mL CAM for 24 h. After washing, resting *A. fumigatus* conidia were added at a multiplicity of infection (MOI) of 0.3 and co-incubated for 24 h before fixation with Mildform and staining. Scale bar = 200 µm. **(B)** Quantification of hyphal area based on fluorescence imaging using hybrid cell count analysis. CAM treatment significantly reduced hyphal area expansion in *M. avium*-infected THP-1 macrophages (****P < 0.0001). Bar graph shows the mean and standard deviation of technical replicates (*n* = 16). Data represent of at least three independent experiments.

Representative fluorescence microscopy images demonstrate *A. fumigatus* hyphae after the co-culture with THP-1 macrophages treated with clarithromycin (**Figure 3A**). Quantitative analysis showed that clarithromycin treatment significantly reduced hyphal area in *M. avium*-infected THP-1 cells compared with untreated infected controls (**Figure 3B**). Clarithromycin administration markedly decreased intracellular *M. avium* burden in THP-1 macrophages (**Supplementary Figure 4A**). In contrast, clarithromycin exposure in uninfected THP-1 cells did not alter fungal growth or enhance antifungal activity against *A. fumigatus* (**Supplementary Figure 4B**). Together, these findings indicate that the improvement in antifungal activity following clarithromycin treatment is attributable to a reduction in intracellular *M. avium* rather than a direct antifungal effect of the drug.

### 3.5 Infection with *M. avium* reduces Dectin-1 expression in THP-1 cells

**Figure 4:**
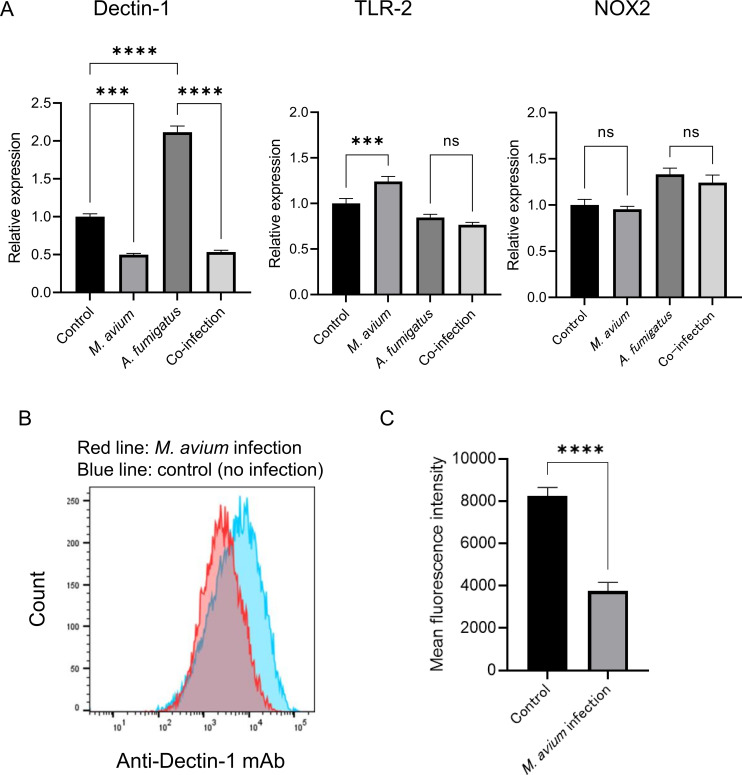
Effect of *Mycobacterium avium* infection on Dectin-1 expression in THP-1 macrophages. **(A)** Quantitative polymerase chain reaction (qPCR) analysis of gene expression in THP-1 macrophages. Expression of selected immune-related genes was compared across four conditions: uninfected control, *M. avium* infection, *Aspergillus fumigatus* infection, and co-infection. Relative expression level was calculated using the ΔΔCT method with the uninfected control normalized to 1 (***P = 0.0003, ****P < 0.0001). Bar graph shows the mean and standard deviation of technical replicates (*n* = 3). Data represents at least three independent experiments. **(B)** Flow cytometry analysis of Dectin-1 surface expression in THP-1 macrophages. The red shaded region represents *M. avium*-infected THP-1 cells, while the blue shaded region represents uninfected control cells. **(C)** Quantification of mean fluorescence intensity (MFI) of Dectin-1 determined by flow cytometry. *M. avium* infection significantly decreased Dectin-1 surface expression compared with uninfected control cells (****P < 0.0001). Bar graph shows the mean and standard deviation of technical replicates (*n* = 5). Data represent at least three independent experiments.

Gene expression analysis showed a significant reduction in Dectin-1 transcript levels in THP-1 macrophages co-infected with *M. avium* and *A. fumigatus* compared with *A. fumigatus* infection alone (**Figure 4A**). Although *M. avium* infection led to an increase in TLR-2 expression, no significant differences in TLR-2 or NOX2 expression were observed when comparing *A. fumigatus* infection with co-infection conditions. Flow cytometry demonstrated that surface expression of Dectin-1 on THP-1 cells was decreased following *M. avium* infection compared with uninfected controls (**Figures 4B, C**). Cell viability did not differ between conditions (**Supplementary Figure 5**), indicating that the reduction in Dectin-1 expression was not attributable to cytotoxicity.

### 3.6 Secondary metabolites from *A. fumigatus* reduce phagocytosis of *M. avium* by THP-1 cells

**Figure 5:**
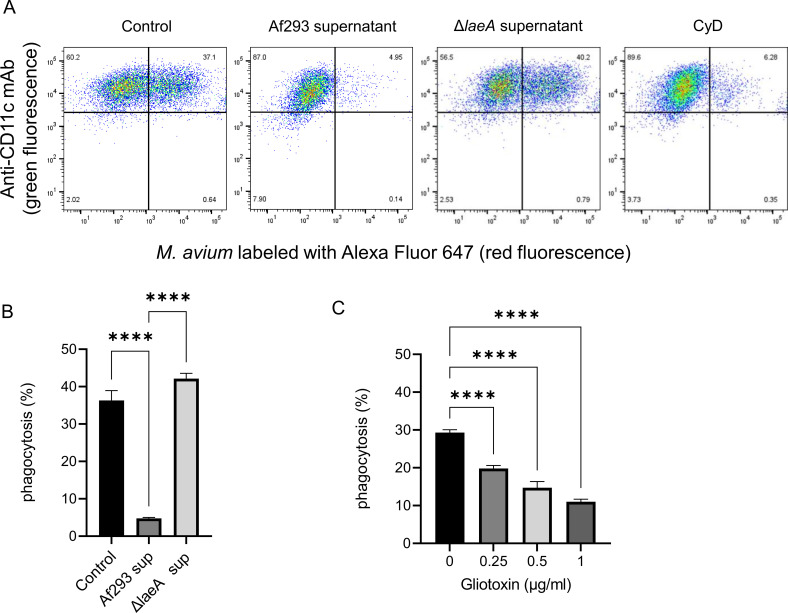
Effect of *Aspergillus fumigatus* secondary metabolites on phagocytosis of *Mycobacterium avium* by THP-1 cells. **(A)** Flow cytometry plots showing phagocytosis of Alexa Fluor 647-labeled *M. avium* by THP-1 macrophages. THP-1 cells were exposed to supernatant from the *A. fumigatus* Af293 strain or the Δ*laeA* mutant strain (deficient in secondary metabolite production) for 30 min prior to co-culture with *M. avium* for 3 h. The upper-right quadrant represents THP-1 macrophages that internalized *M. avium*. Cytochalasin D (CyD) was used as a positive control for inhibition of phagocytosis. **(B)** Quantification of phagocytosis rate. Exposure to Af293 supernatant significantly reduced the phagocytic activity of THP-1 macrophages against *M. avium*, whereas Δ*laeA* supernatant did not alter phagocytosis (****P < 0.0001). Bar graph shows the mean and standard deviation of technical replicates (*n* = 4). Data represent at least three independent experiments. **(C)** Flow cytometry analysis of THP-1 macrophages exposed to increasing concentrations of gliotoxin for 30 min before co-culture with Alexa Fluor 647-labeled *M. avium* 3 h. Bar graph shows the mean and standard deviation of technical replicates (*n* = 4). Data represent at least three independent experiments.

Flow cytometry analysis demonstrated a reduction in *M. avium* phagocytosis by THP-1 macrophages following exposure to culture supernatant from *A. fumigatus* strain Af293 (**Figures 5A, B**). In contrast, THP-1 cells exposed to supernatants from the Δ*laeA* mutant, which is deficient in secondary metabolite production, did not exhibit reduced phagocytic activity compared with untreated controls. Exposure to purified gliotoxin resulted in a concentration-dependent decrease in phagocytosis of *M. avium* by THP-1 cells (**Figure 5C**). No cytotoxicity effect was observed at concentrations of ≤ 1 μg/mL (data not shown).

### 3.7 Prior *M. avium* infection delays clearance of *A. fumigatus* in mouse lungs

**Figure 6:**
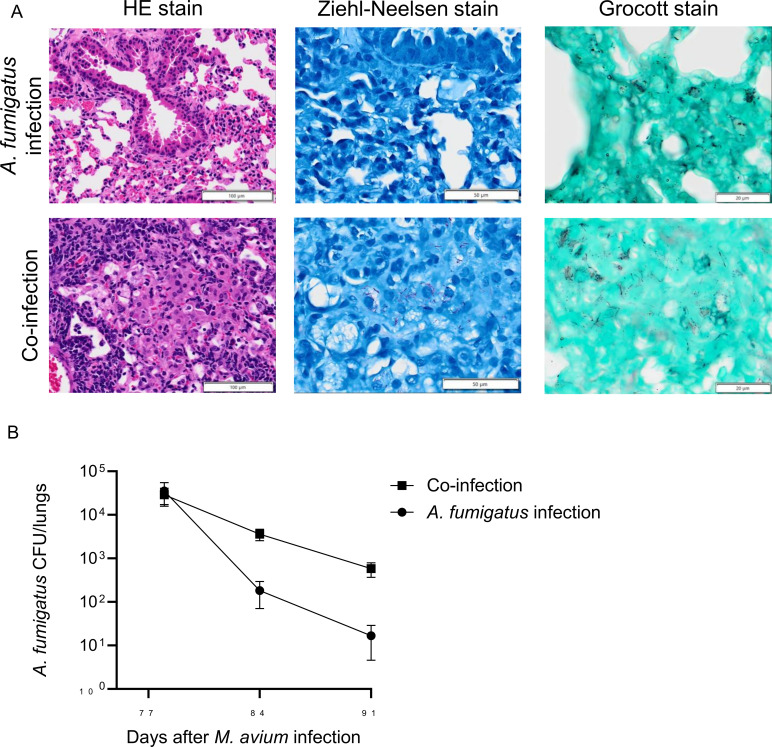
Effect of prior *Mycobacterium avium* infection on clearance of *Aspergillus fumigatus* in mouse lungs. **(A)** Histopathology of mouse lungs 1 day after aspiration of *A. fumigatus* conidia. Mice were intratracheally infected with *M. avium* 11 weeks before fungal challenge; phosphate-buffered saline (PBS)-treated mice served as uninfected controls. Sections were stained with hematoxylin and eosin (H&E) to assess tissue response and Ziehl-Neelsen staining to visualize acid-fast bacilli. Grocott’s methenamine silver staining was used to identify *A. fumigatus* structures. Scale bars = 100 µm, 50 µm, and 20 µm for H&E, Ziehl-Neelsen, and Grocott’s methenamine silver staining, respectively. **(B)** Quantification of fungal burden in mouse lungs. Lungs harvested 1 day (Day 78), 1 week (Day 84), and 2 weeks (Day 91) after *A. fumigatus* aspiration were homogenized, and 50 µL of homogenate was plated onto potato dextrose agar and incubated at 37 °C for 24 h to determine colony-forming units (CFU). Data represent at least two independent experiments. Error bars indicate standard deviation of biological replicates (*n* = 6).

Histopathological analysis using Grocott’s methenamine silver staining demonstrated that *A. fumigatus* was present predominantly in conidial form in *A. fumigatus* infectionmice, whereas extensive hyphal structures were observed in co-infection mice (**Figure 6A**). Colonies of *M. avium* remained detectable in lung tissue harvested on Day 77, confirming persistence of infection throughout the experimental period (data not shown). Quantification of fungal burden showed a significant delay in *A. fumigatus* clearance in the co-incectiongroup compared with *A. fumigatus* infection group(**Figure 6B**).

## 4 Discussion

This study demonstrates a bidirectional interaction between NTM and *A. fumigatus*, whereby NTM promote fungal biofilm formation and impair host antifungal defense mechanisms. Culture supernatants from NTM enhanced *A. fumigatus* biofilm biomass and ECM production and reduced susceptibility to voriconazole. In parallel, infection of macrophages with *M. avium* suppressed phagocytosis of *A. fumigatus*, accompanied by a reduction in surface expression of Dectin-1. Given that Dectin-1 is a key pattern recognition receptor involved in fungal uptake and inflammatory signaling (Steele et al., 2005; Werner et al., 2009), reduced expression is likely to contribute to impaired clearance of *A. fumigatus*. Notably, antimycobacterial treatment restored macrophage antifungal activity, indicating that controlling intracellular NTM infection may indirectly facilitate fungal clearance.

Biofilm is known to influence fungal colonization. *A. fumigatus* biofilms consist of intertwined hyphae embedded in the ECM, contributing to immune evasion and reduced susceptibility to antifungal drugs (Mowat et al., 2008; Kernien et al., 2017). In this study, NTM supernatants increased ECM density and biofilm thickness, correlating with reduced susceptibility to voriconazole. Early biochemical characterization indicates that the active component in the NTM supernatant is heat-stable, < 3 kDa in size, and non-proteinaceous, suggesting the involvement of a small molecule mediator. Further fractionation and analytical work will be necessary to identify this factor.

Previous reports examining fungal–bacterial interactions have described suppressive effects of *S. pneumoniae*, *K. pneumoniae*, *P. aeruginosa*, and *S. maltophilia* on *A. fumigatus* biofilms (Mowat et al., 2010; Melloul et al., 2016; Iwahashi et al., 2020; Curtis et al., 2023). These contrasting findings suggest that NTM-fungal interactions may operate through mechanisms distinct from those observed with common airway bacteria. Geurts et al. (2019) previously reported that *M. avium* supernatants enhanced *A. fumigatus* growth, whereas *M. abscessus* supernatants suppressed it. Their assessment relied on OD measurements, which are less reliable for filamentous fungi, potentially explaining the methodological discrepancy with our findings.

Innate immune mechanisms, particularly those mediated by alveolar macrophages and neutrophils, are critical for early defense *A. fumigatus* (Park and Mehrad, 2009). Dectin-1 recognition of β-D-glucan—a component of the fungal cell wall—initiates cytokine signaling, phagocytosis, and reactive oxygen species production (Gantner et al., 2003) (Steele et al., 2005) (Schorey and Lawrence, 2008). The observed downregulation of Dectin-1 following *M. avium* infection may therefore attenuate macrophage recognition and phagocytic clearance of *A. fumigatus*, contributing to persistent fungal colonization in the setting of NTM-PD.

The role of Dectin-1 in NTM infection remains uncertain. Some studies have reported that Dectin-1 recognizes NTM-derived ligands in addition to β-D glucan (Yadav and Schorey, 2006; Shin et al., 2008), whereas others have shown that *M. abscessus* infection proceeds independently of Dectin-1 signaling (Ochoa et al., 2023). In contrast to our findings, lipopolysaccharide stimulation has been shown to increase Dectin-1 expression in THP-1 cells (Rogers et al., 2013), suggesting that NTM may regulate Dectin-1 through mechanisms distinct from those triggered by other bacterial species. These observations indicate that modulation of Dectin-1 during NTM infection may involve unique host–pathogen interactions. Further mechanistic studies are required to elucidate the underlying pathways and to clarify the functional significance of Dectin-1 downregulation in macrophages during NTM infection *in vivo*.

Secondary metabolites produced by *A. fumigatus* are known to modulate immune function, with effects reported on macrophages and neutrophils (Ye et al., 2021). Gliotoxin, one of the best characterized mycotoxins, has previously been shown to inhibit phagocytosis in murine macrophage-like cells (Schlam et al., 2016). Consistent with this, our findings demonstrate that gliotoxin and other *A. fumigatus*-derived secondary metabolites reduce the ability of macrophages to internalize NTM, suggesting a reciprocal suppression of host immunity that may facilitate colonization of both organisms.

Our study demonstrated that antimycobacterial treatment against *M. avium* improves the antifungal activity of macrophages *in vitro*. This finding suggests that antimycobacterial treatment against NTM-PD may prevent the onset of CPA or inhibit its progression. However, large gaps remain in applying this insight to clinical practice. Future work requires evaluating the antimycobacterial treatment efficacy against NTM in co-infection mouse models and analyzing patient data through clinical studies.

A strength of this study is the integrated assessment of microbial interactions, host immune response, and antifungal susceptibility. To the best of our knowledge, this is the first report demonstrating suppression of Dectin-1 expression in macrophages during *M. avium* infection and showing that antimycobacterial therapy can restore macrophage-mediated fungal clearance. These findings provide a mechanistic rationale supporting the clinical observation that co-infection with NTM and *A. fumigatus* is associated with poor outcomes and highlight antimicrobial therapy as a potential strategy not only for treating NTM-PD but also for improving fungal control.

This study has some limitations. First, the active component in NTM supernatants responsible for promoting *A. fumigatus* biofilm formation remains unidentified. Second, our gene panel was limited to selected mediators; thus, broader transcriptomic analyses are warranted to clarify the immune pathways altered during co-infection. Third, heat-killed conidia were used in qPCR and phagocytosis assays. Therefore, our experiment may not have reproduced the immune responses. Fourth, this study used cell lines and did not confirm the results using macrophages differentiated from normal monocytes. Fifth, our study did not examine whether *Aspergillus* spp. other than *A. fumigatus* affect NTM growth. Further research is needed to determine whether the results of our experiment apply only to specific species. Sixth, we evaluated the expression of Dectin-1 on the surface of THP-1 cells by flow cytometry under only two conditions: *M. avium* infection and uninfected control. Four conditions are required to more securely support the qPCR results. Finally, although our *in vivo* model demonstrated delayed fungal clearance following *M. avium* infection, the detailed immune mechanisms underlying this phenomenon could not be fully delineated.

In conclusion, NTM enhances the growth and colonization of *A. fumigatus* by promoting biofilm development and suppressing macrophage antifungal function, partly through downregulation of Dectin-1. Conversely, *A. fumigatus*-derived secondary metabolites, such as gliotoxin, impair macrophage control of NTM, suggesting a mutually permissive relationship between these pathogens. Understanding the bidirectional interaction between NTM and *A. fumigatus* may inform new therapeutic strategies for managing co-infection in chronic lung disease.

## Data Availability

The raw data supporting the conclusions of this article will be made available by the authors, without undue reservation.

## Glossary

COPD: chronic obstructive pulmonary disease
CPA: chronic pulmonary aspergillosis
NTM-PD: non-tuberculous mycobacterial pulmonary disease
NTM: non-tuberculous mycobacteria
PBS: phosphate-buffered saline
RPMI: Roswell Park Memorial Institute
FBS: fetal bovine serum
OD: optical density
CLSM: confocal laser scanning microscopy
TEM: transmission electron microscopy
qPCR: quantitative polymerase chain reaction*;* TLR-2, toll-like receptor 2
GAPDH: glyceraldehyde-3-phosphate dehydrogenase
MOI: multiplicity of infection
