# Supplementary material for: Immune interaction between Aspergillus fumigatus and non-tuberculous mycobacteria
Source: Front Cell Infect Microbiol. 2026 May 4;16:1810773. doi: 10.3389/fcimb.2026.1810773 (PMC13180838; doi:10.3389/fcimb.2026.1810773)
Supplement: Supplementary file 1 [file SupplementaryFile1.docx]

Supplementary Material

# Supplementary information

**1. Materials and methods**

**1.1 Crystal violet biofilm assay**

As a heat treatment, non-tuberculous mycobacteria (NTM) supernatant was heated at 95 °C for 15 min. NTM supernatant was ultrafiltrated using a 3 kDa Amicon® Ultra (Merck, Darmstadt, Germany) spin filter at 4000×g for 45 min. Voriconazole (Pfizer, New York, NY, USA) was adjusted to a final concentration of 0.125 μg/mL. Its antifungal effect was calculated using the following formula: Antifungal effect (%) = (1 - X/Y) × 100, where X represents optical density (OD) under the conditions with voriconazole, and Y represents OD under the conditions with NTM supernatants or medium control, without voriconazole.

**1.2 Microscopy of biofilms**

Samples for a transmission electron microscopy were fixed in 2.5% glutaraldehyde (TAAB, Berks, UK) solution buffered to pH 7.4 with 0.1M phosphate buffer for 4 h at 4 ℃. Fixation was performed with a 1% osmium tetroxide (Heraeus chemical, Johannesburg, South Africa) solution buffered to pH 7.4 with the same buffer for 2 h at 4 ℃. They were dehydrated in graded series of ethanol (FUJIFILM Wako, Osaka, Japan) and embedded in Epon 812(TAAB). Ultrathin sections were cut with an ultramicrotome (Ultracut S, Leica, Austria) with diamond knife, doubly stained with uranyl acetate and lead nitrate, and observed with an electron microscope (JEM-1200EX, JEOL, Japan) at an accelerating voltage of 80 kV.

**1.3 Evaluation of NTM growth**

*Mycobacterium avium* and *M. abscessus* suspensions were adjusted to the OD of 0.1 at 600 nm in 7H9 medium (Becton Dickinson, Franklin Lakes, NJ, USA). Subsequently, 3.6 mL of the NTM suspension was added to 0.4 mL of *A. fumigatus* supernatant and cultured at 37 °C and 250 rpm. At each evaluation point, 100 μL of the bacterial suspension was transferred to a 96-well plate and growth of NTM was assessed by measuring the OD at 600 nm.

**1.4 Evaluation of intracellular *M. avium* burden in THP-1 macrophages**

After co-culture, extracellular *M. avium* was removed by washing twice with phosphate-buffered saline (PBS). THP-1 macrophages were cultured for 24 hours in RPMI 1640 medium containing 10% fetal bovine serum and clarithromycin (4–16 µg/mL). Subsequently, the supernatant was removed, and the cells were washed twice with PBS. THP-1 macrophages were lysed with ice-cold distilled water containing 0.1% polyoxyethylene(10) octylphenyl ether (FUJIFILM Wako) at 25 °C for 20 min. The cell lysate was diluted with PBS; 20 μL of the lysate was inoculated into 7H10 medium and incubated at 37 °C for 1 week to evaluate the intracellular *M. avium* burden.

**1.5 Viability of THP-1 macrophages**

The viability of THP-1 macrophages was assessed using the 2,3-(bis2-methoxy-4-nitro-5-sulfophenyl)-2H-tetrazolium-5-carboxyanilide (XTT) assay kit (Sigma-Aldrich, St. Louis, MO, USA). Supernatant was removed 24 h after infection with *M. avium*, and XTT reagent was added to THP-1 macrophages at a final concentration of 0.3 mg/mL. The macrophages were then incubated for 1 h at 37 °C in dark area. Subsequently, 100 μL of supernatant was transferred to a 96-well plate, and the OD was measured at 450 nm with a 620 nm reference wavelength. The OD of *M. avium* infection was divided by the OD of medium control (non-NTM infection condition) to determine the cell viability. The cell viability was presented with medium control as 100%.

**1.6 Analysis of flow cytometry**

### 1.6.1 Phagocytosis assay of *A. fumigatus*

Swollen conidia were stained with calcofluor white and incubated with THP-1 macrophages infected with *M. avium* for 2h (multiplicity of infection: 2). After co-incubation, extracellular conidia were washed and removed, and THP-1 macrophages were harvested using 0.25% trypsin-EDTA (FUJIFILM Wako). THP-1 macrophages were stained with a fluorescent anti-CD11c antibody for flow cytometry analysis. Cytochalasin D (Sigma-Aldrich) was used at final concentration of 2 μg/ml.

### 1.6.2 Phagocytosis assay of *M. avium*

THP-1 macrophages were exposed to *A. fumigatus* supernatant and different concentrations of gliotoxin for 30 min. *A. fumigatus* supernatant was diluted 2-fold in RPMI medium before use. Gliotoxin was dissolved in Dimethyl sulfoxide (DMSO), the final DMSO concentration was adjusted to 0.2%. *M. avium* was suspended in 0.1 M sodium bicarbonate solution and incubated with Alexa Fluor 647 NHS ester (Lumiprobe, Hunt Valley, MD, USA) at a concentration of 10 μg/mL. After incubation in dark area for 15 min, *M. avium* was washed twice with PBS and then co-cultured with THP-1 macrophages for 3 h. After co-incubation, extracellular *M. avium* was washed and removed, and THP-1 macrophages were harvested using 0.25% trypsin-EDTA. THP-1 macrophages were stained with a fluorescent anti-CD11c antibody for flow cytometry analysis.

### 1.6.3 Dectin-1 expression in THP-1 macrophages

Dectin-1 expression on the surface of THP-1 macrophages was assessed 24 h after *M. avium* infection. Dectin-1 expression levels were quantified using mean fluorescence intensity.

# Supplementary Figures and Tables

## Supplementary Table 1

**List of Primers used in quantitative polymerase chain reaction**

| Genes | Forward | Reverse |
| --- | --- | --- |
| *GAPDH* | TGCACCACCAACTGCTTAGC | GGCATGGACTGTGGTCATGAG |
| *Dectin‑1* | AGCCTACCTGTAGGTCGACAA | CTGAGGTCAAGATAAATGCAGAAA |
| *TLR-2* | GCAAGCTGCGGAAGATAATG | CGCAGCTCT CAGATTTACCC |
| *NOX2* | CAAGATGCGTGGAAACTACC | TTG AGAATGGATGCGAAGG |

GAPDH, glyceraldehyde-3-phosphate dehydrogenase; TLR-2, Toll-like receptor 2; NOX2, NADPH oxidase 2

## Supplementary Figures

#
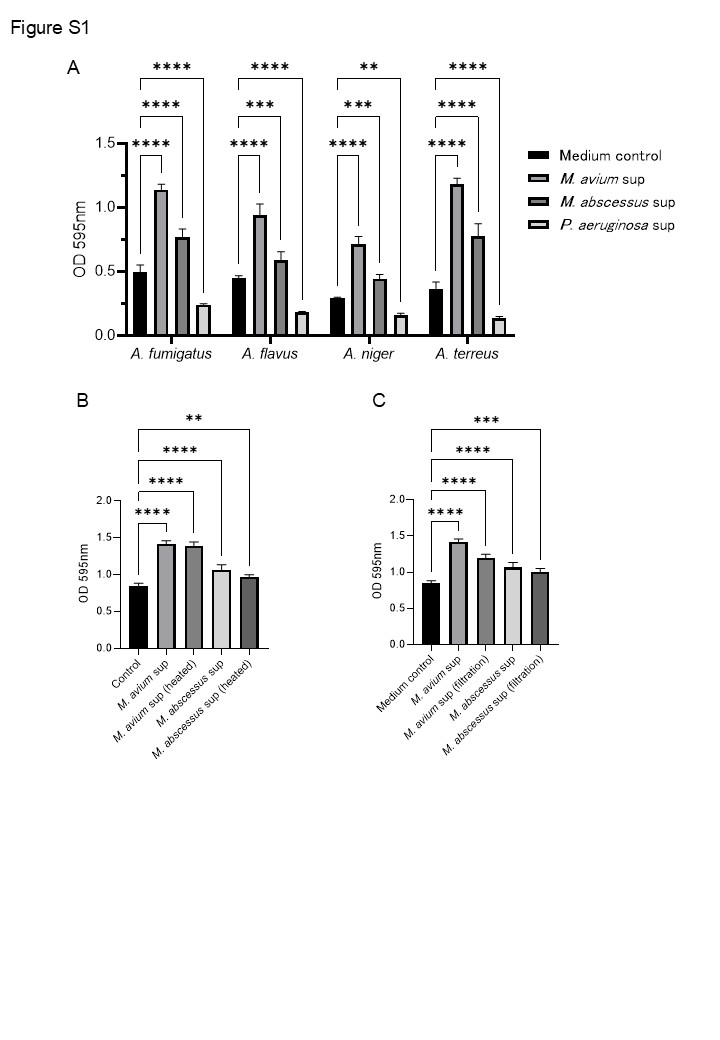


**Supplementary Figure S1. Crystal violet assay assessing biofilm formation by *Aspergillus* species in response to non-tuberculous mycobacteria (NTM) supernatants.**

(A) Quantification of biofilm biomass of *Aspergillus fumigatus,* *A. flavus*, *A. niger,* and *A. terreus* following exposure to NTM culture supernatants. Biofilm formation increased in response to NTM supernatants and decreased following exposure to *Pseudomonas aeruginosa* supernatant (***P* < 0.0021, ****P* < 0.0003, *****P* < 0.0001). Bar graph shows the mean and standard deviation of technical replicates (*n*=4). Data represent at least three independent experiments.

(B) Crystal violet assay performed using heat-treated NTM supernatants. A retained biofilm-promoting effect was observed following heat treatment (***P* < 0.0014, *****P* < 0.0001). Bar graph shows the mean and standard deviation of technical replicates (*n*=8). Data represent at least three independent experiments.

(C) Crystal violet assay performed using ultrafiltrated NTM supernatant (****P* = 0.0001, *****P* < 0.0001). Bar graph shows the mean and standard deviation of technical replicates (*n*=8). Data represent at least three independent experiments.


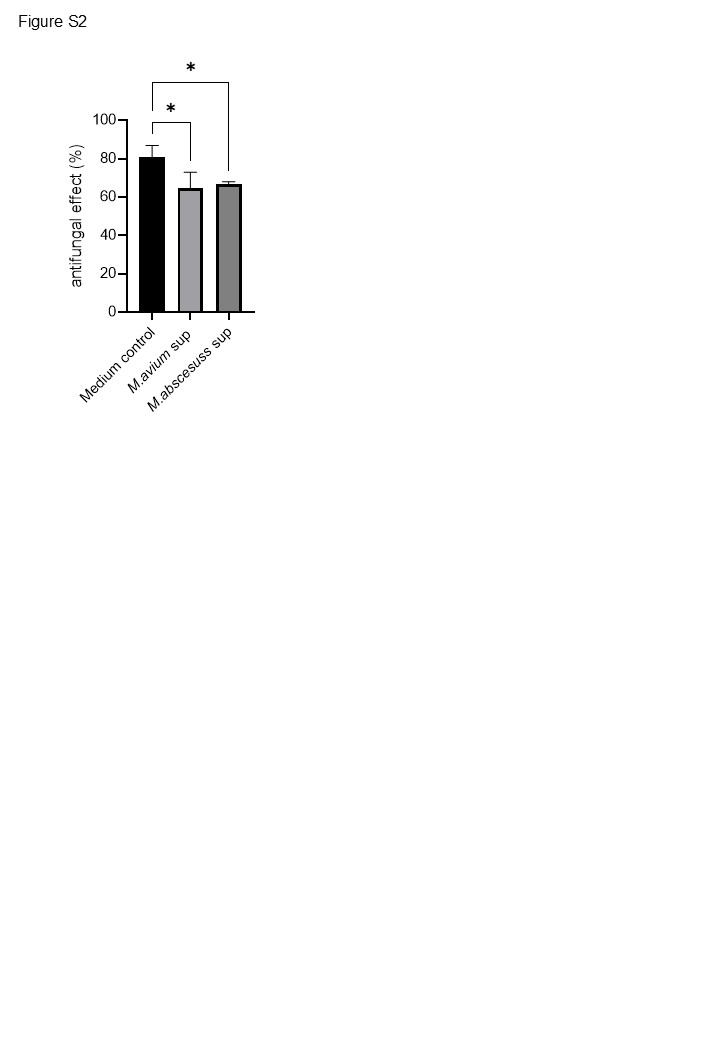


**Supplementary Figure S2. Effect of voriconazole on *Aspergillus fumigatus* biofilm formation in the presence of non-tuberculous mycobacteria (NTM) supernatants.**

Voriconazole susceptibility was evaluated using a modified crystal violet assay in which NTM supernatants were mixed 1:1 with *A. fumigatus* suspensions. The final conidial concentration was adjusted to 1.0 × 10⁵ conidia/mL and the final voriconazole concentration to 0.125 µg/mL. Antifungal activity reflects the reduction in biofilm biomass relative to conditions without voriconazole (**P* < 0.03). Bar graph shows the mean and standard deviation of technical replicates (*n*=4). Data represent at least three independent experiments.
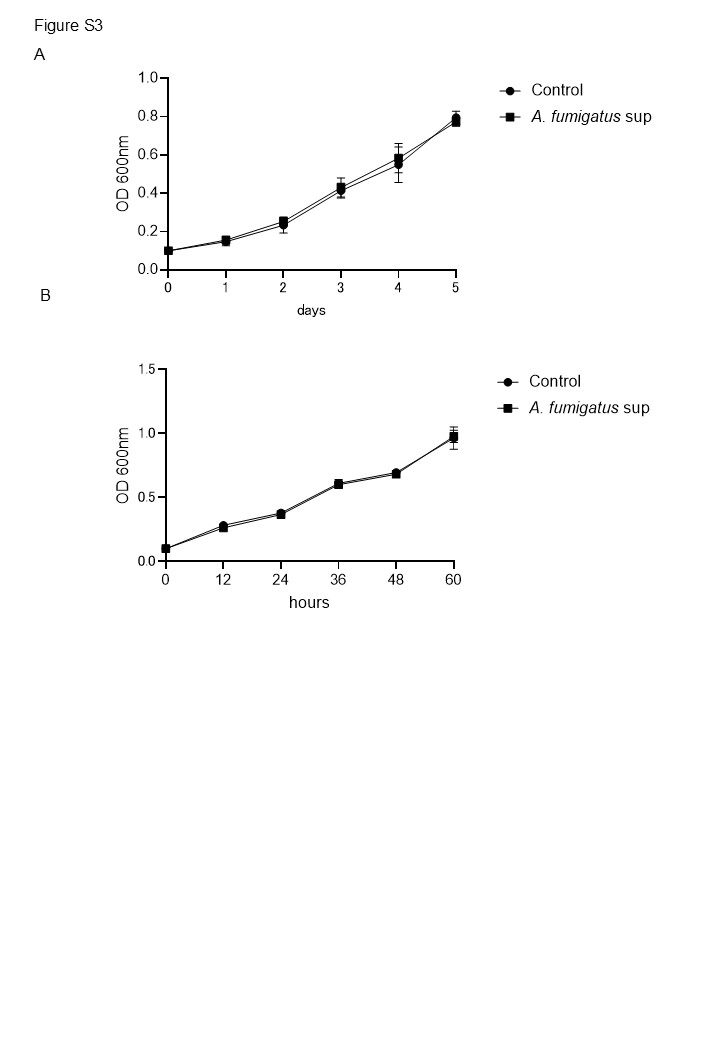


**Supplementary Figure S3. Effect of *Aspergillus fumigatus* supernatant on the growth of non-tuberculous mycobacteria (NTM).**

Suspensions of NTM was adjusted to an optical density (OD) of 0.1 at 600 nm in Middlebrook 7H9 medium. Each suspension (3.6 mL) was mixed with either 0.4 mL of *A. fumigatus* culture supernatant or 0.4 mL RPMI medium control medium.

(A) Growth curve of *Mycobacterium avium*. Bar graph shows the mean and standard deviation of technical replicates (*n*=4). Data represent at least three independent experiments.

(B) Growth curve of *M. abscessus*. Bar graph shows the mean and standard deviation of technical replicates (*n*=4). Data represent at least three independent experiments.
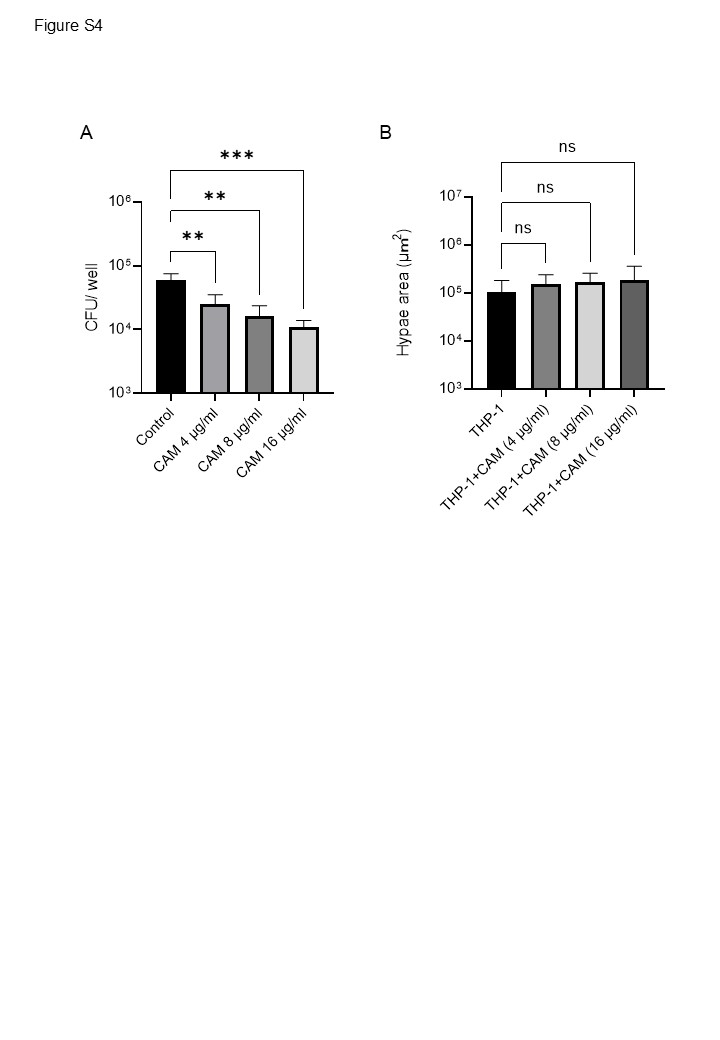


**Supplementary Figure S4**. **Effect of clarithromycin treatment on intracellular *Mycobacterium avium* burden and antifungal activity of THP-1 macrophages.**

(A) Intracellular bacterial burden of *M. avium* following clarithromycin treatment. THP-1 macrophages infected with *M. avium* were treated with clarithromycin, lysed, and plated onto Middlebrook 7H10 agar. Colony-forming units (CFU) per well were quantified (** *P* < 0.005, *** *P* = 0.0005). Bar graph shows the mean and standard deviation of technical replicates (Control and CAM 4µg/mL; *n*=4, CAM 8µg/mL and CAM 16µg/mL; *n*=3). Data represent at least three independent experiments.

(B) Effect of clarithromycin (CAM) on antifungal activity in uninfected THP-1 macrophages. THP-1 macrophages were exposed to CAM (4–16 µg/mL) for 24 h, washed, and co-cultured with resting *Aspergillus fumigatus* conidia for 24 h. Following fixation with Mildform and calcofluor white staining (10 min), hyphal area was quantified using hybrid cell count analysis. Bar graph shows the mean and standard deviation of technical replicates (*n*=16). Data represent at least three independent experiments.


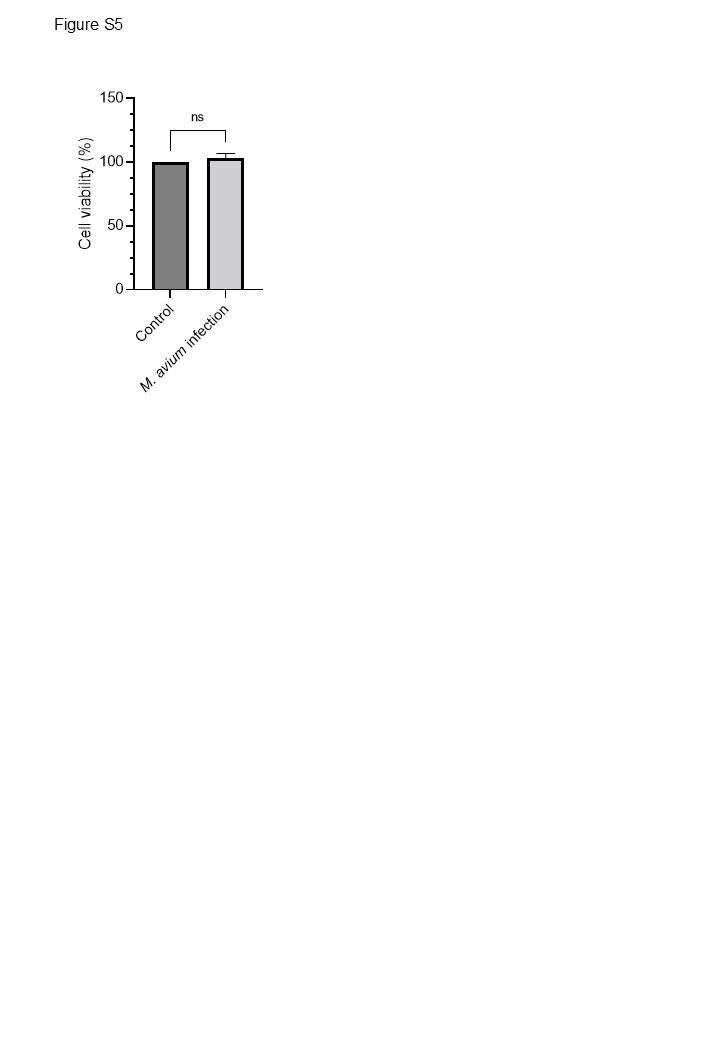


**Supplementary Figure S5. Effect of *Mycobacterium avium* infection on THP-1 macrophage viability.**

Cell viability was assessed 24 h after infection with *M. avium* using an XTT assay (2,3-bis(2-methoxy-4-nitro-5-sulfophenyl)-2H-tetrazolium-5-carboxanilide). Viability is expressed relative to uninfected THP-1 control cells, which were defined as 100%. Bar graph shows the mean and standard deviation of technical replicates (*n*=5). Data represent at least three independent experiments.
